# Clinical Applicability of Ultrasound Shear Wave Elastography in Patients under Hypoglossal Nerve Stimulation Therapy

**DOI:** 10.3390/diagnostics13233493

**Published:** 2023-11-21

**Authors:** Philipp Arens, Thomas Fischer, Ingo Fietze, Thomas Penzel, Steffen Dommerich, Heidi Olze, Markus Herbert Lerchbaumer

**Affiliations:** 1 Department of Otorhinolaryngology, Berlin Institute of Health, Charité—Universitätsmedizin Berlin, Corporate Member of Freie Universität Berlin, Humboldt-Universität zu Berlin, 10117 Berlin, Germany; steffen.dommerich@charite.de (S.D.); heidi.olze@charite.de (H.O.); 2 Department of Radiology, Berlin Institute of Health, Charité—Universitätsmedizin Berlin, Corporate Member of Freie Universität Berlin, Humboldt-Universität zu Berlin, 10117 Berlin, Germany; thom.fischer@charite.de (T.F.); markus.lerchbaumer@charite.de (M.H.L.); 3 Interdisciplinary Center of Sleep Medicine, Berlin Institute of Health, Charité—Universitätsmedizin Berlin, Corporate Member of Freie Universität Berlin, Humboldt-Universität zu Berlin, 10117 Berlin, Germany; ingo.fietze@charite.de (I.F.); thomas.penzel@charite.de (T.P.)

**Keywords:** obstructive sleep apnea, hypoglossal nerve stimulation therapy, sonography, ultrasound, shear wave elastography, phenotyping

## Abstract

Relationship between stiffness of genioglossi (GG) and geniohyoidei (GH) muscles under electric hypoglossal nerve stimulation therapy (HNS) in relation to success of therapy was investigated with additional special focus on tongue movement. Patients and Methods: Clinical and sleep laboratory parameters of a cohort of 18 patients with known shear wave velocity (SWV) data of the ipsilateral and contralateral musculi GG and GH (sGG, sGH and nGG, nGH) before and under HNS therapy were analyzed. The SWV was already determined using the ultrasonic shear wave elastography (US-SWE) technique. Results: Median Epworth Sleepiness Scale (ESS) was 8 (IQR 12), median baseline Apnoe–Hypopnoe Index (AHI) 31.65 (IQR 25.1), median AHI under HNS therapy 16.3 (IQR 20.03). Therapy success: 9/18 patients (AHI during therapy < 15/h). There was no significant difference in SWV (sGG, sGH, nGG and nGH) between therapy responders and non-responders during therapy. Also, no difference could be seen with respect to the difference and increase in SWV values without and with stimulation. Examination of SWV values (sGG, sGH, nGG, nGH during stimulation, difference of SWV values stimulation − no stimulation, increase factor of SWV) revealed a significant negative correlation between the AHI under therapy and the measured SWV of the musculus GH of the contralateral side during stimulation (−0.622, *p* = 0.006). Patients with bilateral protrusion of the tongue differed regarding to therapy success in increase in SWV in sGG (*p* = 0.032). Tongue protrusion to contralateral: A significant difference between patients with AHI during therapy < 15/h in SWV values at sGG without stimulation (*p* = 0.021) was seen, with also a correlation to the current AHI under therapy (*p* = 0.047) and the change factor of the AHI (*p* = 0.015). Conclusion: Stiffness of the target muscle does not appear to be an isolated measure of the success of HNS therapy. This observation may have implications for future decision-making processes in the process of titrating electrical therapy parameters. But the technique of US-SWE may be useful for future research of the neurophysiology of the tongue and OSA phenotyping.

## 1. Introduction

**Figure 1:**
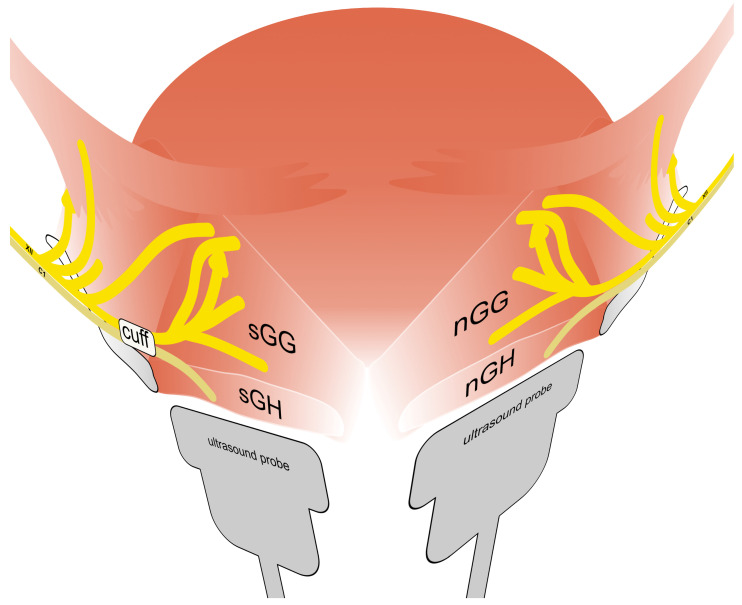
Schematic diagram of the neuroanatomy of the tongue (according to Mu et al. 2010 [10]) including typical position of stimulation electrode (cuff). Shown is the innervation of the extrinsic muscles of the tongue by the nervus hypoglossus (XII), especially genioglossus muscle of the stimulated side (sGG) and the non-stimulated side (nGG). In addition, innervation of the geniohyoid muscle of the stimulated side (sGH) and the non-stimulated side (nGH) through nerve fibers from the first cervical nerve (C1) is illustrated. The position of the ultrasound probe during examination of each side is indicated.

**Figure 2:**
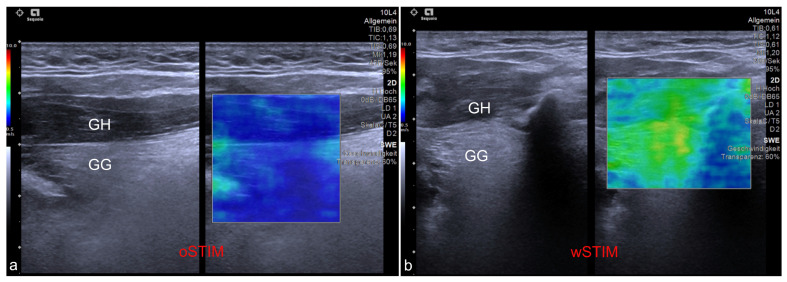
Exemplary ultrasound images showing the application of shear wave elastography at the genioglossus (GG) and geniohyoid (GH) muscles. (**a**) Measurement of a patient without stimulation of the hypoglossal nerve (oSTIM). (**b**) Measurement of a patient during stimulation of the hypoglossal nerve (wSTIM).

Hypoglossal nerve stimulation therapy (HNS) in patients with obstructive sleep apnea (OSAS) is now an established therapy in patients with continuous positive airway pressure (CPAP) therapy intolerance [1]. In the context of OSAS surgery, the therapy represents a functional alternative to the existing resective surgical procedures [2]. Various stimulator models are available on the market. Currently, the most widely used is a respiratory rate-guided system with unilateral distal hypoglossal nerve stimulation (udHNS). In contrast, there are the concepts of unilateral proximal stimulation and bilateral distal stimulation of the nerve without respiratory sensing [3,4,5]. The concept of HNS therapy is based on electrical stimulation of the hypoglossal nerve in order to achieve protrusion of the tongue and stabilization of the upper airway by stiffening the muscles through muscle activation [6]. In the past decade, anatomy, innervation, and function of individual muscle groups of the tongue have been better understood [7,8,9]. Main trunk of the hypoglossal nerve divides into two parts: the lateral (l-XII) and medial (m-XII) branches. M-XII innervate extrinsic tongue-protruding muscle fibers. These are essentially the genioglossus muscle (GG) and the intrinsic stiffening tongue muscles (mainly the transverse (T) and vertical (V) muscles of tongue [8]. Consequently, the goal of implantation of an udHNS system is to stimulate the m-XII branches selectively while excluding the l-XII branches, which innervate extrinsic retractor muscles (basically hyoglossus and styloglossus muscle) [10]. The main target muscle of the therapy is the GG. It is a fan-shaped muscle that originates at the symphysis of the mandible (see also Figure 1 and Figure 2). [11]. GG consists of horizontal fibers (GGh) and oblique fibers (GGo). Contraction of the GGh leads to symmetric pharyngeal enlargement, reducing pharyngeal collapsibility. In contrast, contraction of the GGo is able to compress the tongue and compromise the posterior airspace, which may indicate greater involvement of the airway opening by the GGh fibers [8,12,13]. Another (subordinate) target muscle of udHNS therapy is the geniohyoid muscle (GH), which is innervated by fibers of the first cervical nerve (C1). This nerve is in most cases located close to the hypoglossal nerve and induces (when also stimulated) forward movement of the hyoid, which may improve treatment outcome [14]. In daily routine, the extent and pattern of muscle activation during HNS therapy is only assessed visually and—if necessary—optimized using sleep laboratory titration. This is guided by the polysomnographic parameters. Up to now, the possibility of directly objectifying the effect of stimulation on the target muscles using measurable simple parameters has been lacking in office-based routine practice. However, ultrasound is an excellent tool in the head and neck region to examine the soft tissues of the neck, the floor of the mouth, and the tongue [15]. The technique of ultrasound shear wave elastography (US-SWE) to study the tongue is promising in many ways. On the one hand, with regard to the practical use for the evaluation of patients under HNS therapy, on the other hand, to gain insights into the neurophysiology of the tongue beyond HNS therapy. Only a few studies have been published that address the issue of elastography in patients with OSAS [16,17,18,19,20]. In particular, Brown et al. published a study that generally address tongue stiffness in patients with OSAS compared to a control group. Here, lower values were found in magnetic resonance elastography in the OSAS group [19]. The US-SWE findings of Chu et al. support this observation [18]. Patients with a hypoglossal nerve stimulator are particularly suitable for gaining knowledge about the neurophysiology of the tongue, as the “laboratory” is virtually implanted in these patients (see also Figure 1). Our previously published results are the only ones investigating HNS therapy using US-SWE. We have previously shown that US-SWE is able to objectively measure the stiffness of the GG and GH muscle and that the results differ between electrically stimulated muscle and non-stimulated muscle [16]. The aim of the present study was to assess the clinical outcome of our cohort of patients to find out whether US-SWE can contribute in a simple way to draw conclusions about the therapeutic effect of HNS therapy.

## 2. Materials and Methods

**Figure 3:**
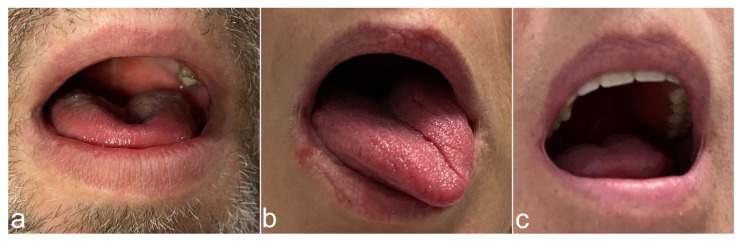
Tongue protrusion patterns during hypoglossal nerve stimulation at the right side. (**a**) Bilateral tongue protrusion. (**b**) Tongue protrusion to contralateral. (**c**) Protrusion pattern that does not fit into the first two categories. This patient presented a movement of the tongue slightly upwards and partly backwards.

A cohort of 18 out of 25 patients with an udHNS system (Inspire Medical Systems, Inc., Minneapolis, MN, USA) implanted an activated at our department between August and November 2020 that agreed to take part at the study. Median age was 62 years (IQR, 55–65). Thirteen patients were male (83.3%). Median body mass index (BMI) was 30.4 kg/m2 (IQR, 27.3–32.1). The US-SWE data were collected as follows and have been published in detail by Arens et al. 2021 [16] with a dedicated description of the technique. Patient position was supine with the head raised. First, gray-scale B-mode US of the tongue and submandibular area was performed in transverse and longitudinal planes for adequate assessment of target muscles. Examinations were performed using a high-end US system with a 4–10 MHz multifrequency linear array transducer and a center frequency of 7 Mhz (Acuson Sequoia, Siemens Healthineers, Erlangen, Germany). Quantitative evaluation of tissue stiffness was acquired by Virtual Touch™ software (Virtual Touch 2D SWE-Small Parts, Software Version VA11 using the ACUSON Sequoia, General software Version 1.0, Siemens Healthineers, Erlangen, Germany) allowing real-time measurement using acoustic radiation force impulse (ARFI) imaging technology. US-SWE examinations were performed in longitudinal to visualize superficial geniohyoid and deeper genioglossal muscles in one image (see also Figure 1 and Figure 2). Two-dimensional SWE approach was used to take repeated SWE measures before and during electrical stimulation with the predefined, patient-adapted parameters (amplitude, frequency, duration of stimulation). Representative stiffness indicated as SWE of each measured muscle without and with stimulation was given as the median of 15 measurements and corresponding interquartile range. Median SWV pre stimulation was 2.11 m/s (IQR, 1.92–2.49) for the sGH muscle and 2.50 m/s (IQR, 2.04–3.00) for the sGG muscle. SWV for the nGh muscle was 2.03 m/s (1.75–2.49) and 2.48 m/s (2.18–2.80) for the nGG muscle pre stimulation. Configuration settings of the stimulator were: “+-+” (bipolar) in 17 patients and “0-0” (unipolar) in one patient. Stimulation duration was 90 ms in 17/18 patients and 60 ms in 1/18 patients. The frequency of stimulation was 33 Hz in all patients. The SWV values of the patients under the individual therapeutic setting were as follows: median SWV in the sGH muscle was 2.53 (IQR, 2.20–3.69) m/s and in the sGG muscle 4.78 (IQR, 4.04–5.36) m/s. At the contralateral side SWV in the nGH muscle was 1.96 (IQR, 1.62–2.09) m/s and at the nGG muscle 2.21 (IQR, 1.73–2.39) m/s. The cohort show following tongue protrusion pattern during stimulation: protrusion to opposite site of stimulation *n* = 8/18 (44.4%), bilateral (symmetric) protrusion *n* = 9/18 (50.0%). One patient showed a tongue protrusion that did not match both patterns [4] (see also Figure 3). Within the scope of this study, the outcome of the udHNS therapy was assessed. The clinical sleep parameters of the patients (apnea, hypopnea index, and Epworth sleepiness scale (ESS)) were collected. If a current polysmonography (PSG) with the tested parameters was available, it was used for analysis. If not, a current PSG at the sleep laboratory or at least polygraphy (PG) (due to the COVID-19 pandemic) with the tested parameters was performed. This study was conducted in accordance with the Declaration of Helsinki and with the approval of the local ethics committee (EA1/263/19). With the clinical sleep parameters collected, we analyzed the extent to which outcome correlated with the measured SWV values of musculi GG and GH and the extent to which clinically relevant group differences could be identified between patients with different tongue protrusion patterns. Continuous variables were tested for normal distribution (Kolmogorov–Smirnov test). Because not all data were normally distributed, variables are reported as median and interquartile range (IQR). Group differences were tested with Mann–Whitney U-test; correlations were examined with Spearman’s rank correlation coefficient. A two-sided significance level of α < 0.05 was defined to indicate statistical significance (p). Due to multiple testing, corrected *p*-values using Bonferroni–Holm’s method are also provided in the tables (corr. *p*). All statistical analyses were performed using SPSS software (IBM Corp., released 2019. IBM SPSS Statistics for Windows, Version 26.0. Armonk, NY, USA: IBM Corp.).

## 3. Results

Clinical data could be collected from all 18 patients (current ESS, baseline AHI, and AHI upon therapy). The current median ESS was 8 (IQR 12). Median baseline AHI before implantation of the HNS system was 31.65 (IQR 25.1). The current median AHI under HNS therapy with the parameters tested was 16.3 (IQR 20.03). The difference between baseline AHI and AHI during therapy was statistically significant (*p* = 0.03). PSG was performed in 11 of these patients and PG in 7. Therapy success was defined as an AHI during therapy < 15/h (therapy success AHI < 15/h). This was the case in 9/18 patients (see also Table 1). No significant difference could be seen between the patients with and without therapy success in terms of SWV (sGG, sGH, nGG and nGH) under therapy. Likewise, no difference could be seen in this context with respect to the difference (DIFF SWV) and increase (change factor SWV) in SWV values without (oSTIM) and with stimulation (wSTIM) (see Table 2). Examination of SWV values (sGG, sGH, nGG, nGH under stimulation, difference of SWV values stimulation-no stimulation, increase factor of SWV) in relation to ESS, the absolute AHI values under therapy and the change of AHI under stimulation compared to baseline (change factor) revealed only a significant negative correlation between the AHI under therapy and the measured SWV of the musculus GH of the contralateral side during stimulation (SWV nGH wStim; −0622, *p* = 0.006). After adjusting the *p*-value with Bonferroni–Holm’s method, *p*-value did not reach significance anymore (corr. *p* = 0.096) (see also Table 3).

### 3.1. Clinical Results According to the Pattern of the Tongue Protrusion

The one patient whose tongue movement could not be classified into “bilateral protrusion” or “protrusion to contralateral” was excluded from the analysis. The current median ESS in patients with bilateral protrusion was 5 (IQR 5) in patients with protrusion to contralateral it was 14.5 (IQR 12). Median baseline AHI before implantation of the HNS system was 26.4/h (IQR 36.3) versus 36.2/h (IQR 20.9). The current median AHI under HNS therapy with the parameters tested was 14.5/h (IQR 13.05) versus 15.1/h (IQR 31.43). There was no difference between the level of ESS and the direction of tongue movement under stimulation therapy. There was also no significant difference in the level of AHI and the change in AHI under therapy. There was also no group difference here with respect to the success of therapy (see Table 4).

### 3.2. US-SWE Results of Bilateral and Contralateral Tongue Protrusion Groups and Their Correlation to Clinical Outcome

A closer look at the SWV results in relation to the direction of tongue protrusion revealed the following results.

- Bilateral protrusion: 5/9 patients with bilateral tongue protrusion were successfully treated (AHI < 15/h). No significant difference could be seen between the patients with and without therapy success in terms of absolute SWV (sGG, sGH, nGG and nGH) (see also Table 5). Patients with bilateral protrusion of the tongue under HNS therapy differed in clinical outcome with respect to the factor of increase in SWV in the genioglossus muscle (at the stimulator side (change factor SWV sGG wSTIMoSTIM, *p* = 0.032). Interestingly, a higher factor of SWV change in the ipsilateral GG muscle was measured in patients with treatment failure here. After adjusting the *p*-value with Bonferroni–Holm’s method, *p*-value did not reach significance value anymore (corr. *p* = 0.384). No further difference could be seen in this context with respect to the difference and increase in SWV values without and with stimulation regarding the other muscles (see also Table 5). Examination of SWV values (sGG, sGH, nGG, nGH under stimulation, increase factor of SWV) in relation to ESS, the absolute AHI values under therapy and the change of AHI under stimulation compared to baseline (change factor) revealed no significant correlation (see also Table 6).
- Protrusion to contralateral: 4/8 patients with tongue protrusion to the contralateral side were successfully treated (AHI < 15/h). A difference could only be seen between the patients with and without therapy success in terms of absolute SWV (sGG, sGH, nGG and nGH) at sGG without stimulation (2.08 (IQR 0.64) vs. 2.97 (IQR 0.51), *p* = 0.029). After adjusting the *p*-value with Bonferroni–Holm’s method *p*-value did not reach significance value anymore (corr. *p* = 0.348). Examination of SWV values (sGG, sGH, nGG, nGH under stimulation, increase factor of SWV) of the patients with tongue protrusion to the contralateral side in relation to ESS, the absolute AHI values under therapy and the change of AHI under stimulation compared to baseline (change factor) revealed also a correlation between SWV values at sGG without Stimulation to the current AHI under therapy (0.714; *p* = 0.047) and the change factor of the AHI (0.810; *p* = 0.015). After adjusting the *p*-value with Bonferroni–Holm’s method *p*-value did not reach significance value anymore (corr. *p* = 0.564 and corr. *p* = 0.18) (see also Table 5 and Table 7).

## 4. Discussion

The results of the present study can be summarized as follows:

- No general connection with the measured US-SWE values at the musculus GG and GH and the therapy outcome under HNS therapy can be found.
- There are indicators of a negative correlation between the AHI under therapy and the measured SWV of the musculus GH of the contralateral side during stimulation.
- The SWV of the genioglossus muscle of the stimulated side seem to differ in terms of clinical success parameters depending on the tongue movement pattern under stimulation.

In our cohort studied using US-SWE of 18 patients under hypoglossal stimulation, we previously showed that SWV as a measure of muscle stiffness under stimulation for the GG and GH muscles was significantly different from stiffness without stimulation [16]. This led to the hope of having found a method with US-SWE that may allow for therapy success estimation in OSAS patients with an implanted hypoglossal nerve stimulation system. However, the values found do not support this thesis. In our cohort, SWV as a simple measure of the stiffness of the GG and GH does not differ between responders and non-responders. Ultimately, this result reflects the experience with hypoglossal stimulation therapy itself. The mere implementation of stimulation therapy does not appear to be a predictor of success. Further factors or preconditions seem to be decisive for the success of the therapy. The fact that the also the level of the AHI does not correlate with the SWV of the GG and GH is surprising in this context but may be due to the small cohort size or the relatively high number of non-responders at our cohort.

The only noticeable difference was a possible negative correlation shown between AHI under therapy and SWV of the contralateral musculus GH of the opposite side, taken in isolated, could of course be an indication that the co-reaction (stiffening) of the muscles of the opposite side under stimulation is helpful for the resolution of the obstructions. Finally, the somewhat newer alternative concept of bilateral hypoglossal stimulation also aims at stimulating both muscles for a good therapy success [5]. However, considering the fact that no corresponding correlations could be shown for both actual target muscles—the musculus GG of each side—and the ipsilateral GH, this seems quite unlikely. The GH muscle is activated by fibers of the first cervical nerve (C1) [8]. Usually, when implanting a udHNS system, an attempt is made to include these fibers in the stimulation (see also Figure 1). Studies have shown that inclusion of the nerve leads to positive clinical effects. However, this inclusion is of course unilateral, so activation of the opposite side under stimulation is unlikely. The SWV results we published for the contralateral GH under stimulation also showed no increase in tongue stiffness, but a decrease in values. This is—mechanically considered—also conclusive for a paired muscle with the same anatomical origin and insertion. The reason why lower SWV values at the GH of the opposite side are possibly associated with a worse clinical outcome would have to be investigated in the future by a simultaneous examination of the airway opening.

The question of why tongue protrusion differs in patients during udHNS remains clinically interesting and exciting from a neurophysiological perspective. Clinically visible are usually two or three predominant movement patterns (see also Figure 3). The expected protrusion of the tongue to the anterior with a movement to the contralateral, a symmetrical bilateral protrusion of the tongue and a mixed activation pattern. In 2020, Heiser et al. studied this phenomenon descriptively electromyographically in more detail. The resulting explanation is a cross innervation of the tongue [21]. Interestingly, we did not find any differences in SWV/tongue stiffness between the ipsi- and contralateral musculi GG and GH with respect to the pattern of tongue protrusion. One possible explanation for this is, on the one hand, that we measured only muscles of the extrinsic tongue musculature and not the intrinsic musculature. However, this does not explain the difference to the study of Heiser et al. This workgroup had demonstrated electromyographically higher potentials at the contralateral GG during bilateral stimulation. In conclusion, it must be stated that the quantitative measurement of tongue stiffness alone does not seem to allow for a statement on the extent and type of movement of the muscle. To further explore the phenomenon and value of cross-innervation, we examined the clinical outcomes of our cohort in relation to tongue protrusion. The only visible difference was that patients with a greater increase in stiffness of the GG muscle during therapy on the stimulator side had worse results in terms of therapy success (AHI < 15/h). However, this was only in the group with bilateral tongue protrusion. A possible explanation would of course be that patients with a worse therapy success are also electrically stimulated at a higher level to improve the outcome. We investigated this and could not find any correlation between stimulation intensity and AHI (0.120; *p* = 0.635) or difference regarding to therapy success in our cohort (1.9 V (IQR 0.85) vs. 2.1 V (IQR 1.25), *p* = 0.931). Furthermore, in patients with tongue protrusion to contralateral¸ we found a difference between the patients with and without therapy success in terms of absolute SWV at sGG without stimulation. This was also shown in the simple correlation to the AHI under therapy and the change of the AHI under therapy. This result in particular is surprising. These results state that a higher muscle stiffness of the muscle before the start of the stimulation and then a movement of the muscle to the opposite side might be associated with a worse therapy outcome. In conclusion, the data we have collected and those we have already published do not provide a reliable explanation for this observation.

A major limitation of this study is the small case number of 18 patients. Due to the many statistical tests performed at this sample, the risk of Type I error is increased in this investigation. Bonferroni–Holm’s correction instead seems to underestimate the significance of some effects due to lower test power when using it.

The distribution of responders and non-responders in our cohort is special, as the response rates of HNS therapy are higher in the literature [22]. The discrepancy may be explained by the fact that only 18 of 25 of our patients took part at this study. Patients with poorer outcomes may be more open to reexamination with medical contact, hoping that this will benefit their previously unfavorable course of therapy. On the other hand, the equal distribution of responders and non-responders was rather advantageous for the statistical analysis of this study. A further limitation was that some patients could only be examined using a PG system due to the ongoing COVID-19 pandemic and the associated restrictions on sleep laboratory capacity. This has an impact on the quality of the AHI values obtained. Other imitations are missing data on the type of airway collapse. Although a drug-induced sleep endoscopy was performed on each patient prior to implantation, it was not standardized in such a way that this data could be used for a meaningful group comparison within this study.

Finally, the data presented here, as well as the previously published results of SWV in relation to tongue movement pattern, illustrates that although the measurement of the velocity of the shear wave at the genioglossus muscle can at this stage tell us something about a general muscle response, it does not seem to be sufficient as a sole measurement to classify the type and effect of the movement. In the future, it is necessary to find out whether the time or the place of the measurement at the muscle is decisive, or whether a relation to other measurement points or muscles (e.g., intrinsic tongue musculature) can better answer clinical observations or neurophysiological questions. At the very best, these studies should be controlled electromyographically, taking into account the observations made by Heiser el al.

Moreover, the results published here are interesting in relation to the concept of OSA phenotyping. Muscle responsiveness of the upper airway muscles is one of the key elements of the PALM concept (Passive Pcrit, Arousal threshold, Loop gain, and upper airway Muscle responsiveness) [23]. In addition, machine learning models have demonstrated their utility for research on cohorts of patients with OSAS [24]. A future approach to constructively integrate SWV values into the diagnosis and treatment of patients could lie in the evaluation of integration into multiparametric assessment and analysis using artificial intelligence.

## 5. Conclusions

Upon overall reflection of the results, the following conclusions can be drawn from our point of view:

- Contrary to expectations, the simple measurement of SWV of the genioglossi and geniohyoidei muscles under HNS therapy is not suitable for drawing conclusions about possible sleep medical therapy success.
- Measured SWV values at the GG and GH muscles do not allow for simple differentiation of the tongue movement pattern under therapy. In the future, neurophysiological studies of the tongue in combination of electromyography with US-SWE investigations could provide valuable insights in both directions.

## Figures and Tables

**Table 1:** Results of clinical therapy outcome parameters.

All Patients (*n* = 18)	
Current ESS	8 (IQR 12)
Baseline AHI (events/h)	31.65 (IQR 25.1)
AHI under therapy (events/h)	16.3 (IQR 20.03)

Abbreviations: Apnoe–Hypopnoe Index (AHI), Epworth Sleepiness Scale (ESS).

**Table 2:** General evaluation of SWV data with regard to therapy success (Mann-Whitney U-test).

All Patients (*n* = 18)	
SWV Variable (Median (Unit), IQR)	Therapy Success AHI < 15/h (Yes vs. No)
SWV sGH oSTIM (2.11 m/s, IQR 0.57)	2.12 (IQR 0.47) vs. 2.10 (IQR 0.69); *p* = 0.931 (corr. *p* ≥ 0.999)
SWV sGH wSTIM (2.53 m/s, IQR 1.5)	2.67 (IQR 1.52) vs. 2.40 (IQR 1.53); *p* = 0.666 (corr. *p* ≥ 0.999)
DIFF SWV sGH wSTIM-oSTIM (0.45 m/s, IQR 1.89)	0.48 (IQR 1.78) vs. 0.41 (IQR 1.84); *p* = 0.605 (corr. *p* ≥ 0.999)
SWV sGG oSTIM (2.50 m/s, IQR 0.96)	2.32 (IQR 1.02) vs. 2.73 (IQR 1.05); *p* = 0.730 (corr. *p* ≥ 0.999)
SWV sGG wSTIM (4.78 m/s, IQR 1.32)	4.98 (IQR 1.64) vs. 4.58 (IQR 1.05); *p* = 0.730 (corr. *p* ≥ 0.999)
DIFF SWV sGG wSTIM-oSTIM (2.16 m/s; IQR 1.39)	1.99 (IQR 1.51) vs. 2.29 (IQR 1.36); *p* = 0.666 (corr. *p* ≥ 0.999)
SWV nGH oSTIM (2.03 m/s, IQR 0.73)	2.15 (IQR 0.48) vs. 1.77 (IQR 0.82); *p* = 0.094 (corr. *p* ≥ 0.999)
SWV nGH wSTIM (1.69 m/s; IQR 0.48)	2.02 (IQR 0.46) vs. 1.76 (IQR 0.46); *p* = 0.05 (corr. *p* = 0.75)
DIFF SWV nGH wSTIM-oSTIM (−0.16 m/s; IQR 0.53)	−0.15 (IQR 0.80) vs. −0.24 (IQR 0.51); *p* = 1.00 (corr. *p* ≥ 0.999)
SWV nGG oSTIM (2.48, IQR 0.62)	2.44 (IQR 0.48) vs. 2.51 (IQR 0.78); *p* = 1.00 (corr. *p* ≥ 0.999)
SWV nGG wSTIM (2.21 m/s; 1.06)	2.26 (IQR 1.96) vs. 1.86 (IQR 1.17); *p* = 0.190 (corr. *p* ≥ 0.999)
DIFF SWV nGG wSTIM-oSTIM (−0.43 m/s; IQR 1.16)	0.11 (IQR 1.66) vs. −0.49 (IQR 1.59); *p* = 0.190 (corr. *p* ≥ 0.999)
Change Factor SWV sGH wSTIMoSTIM (1.24; IQR 0.81)	1.27 (IQR 0.80) vs. 1.22 (IQR 0.90); *p* = 0.546 (corr. *p* ≥ 0.999)
Change Factor SWV sGG wSTIMoSTIM (1.82; IQR 0.64)	1.76 (IQR 0.80) vs. 1.89 (IQR 0.80); *p* = 0.605 (corr. *p* ≥ 0.999)
Change Factor SWV nGH wSTIMoSTIM (0.93; IQR 0.25)	0.94 (IQR 0.35) vs. 0.92 (IQR 0.27); *p* = 0.931 (corr. *p* ≥ 0.999)
Change Factor SWV nGG wSTIMoSTIM (0.82; IQR 0.51)	1.04 (IQR 0.64) vs. 0.77 (IQR 0.57); *p* = 0.094 (corr. *p* ≥ 0.999)

Abbreviations: shear wave velocity (SWV), musculus geniohyoideus at the stimulation side (sGH), musculus geniohyoideus at the non-stimulated side (nGH), musculus genioglossus at the stimulation side (sGG), musculus genioglossus at the non-stimulated side (nGG), with stimulation (wSTIM), without stimulation (oSTIM), difference (DIFF), Apnoe–Hypopnoe Index (AHI), corrected *p*-value using Bonferroni–Holm’s method (corr. *p*).

**Table 3:** Spearman correlations between SWV and clinical therapy outcome parameters.

All Patients (*n* = 18)			
SWV Variable (Median (Unit), IQR)	Correlation to Current AHI under Therapy (16.3/h; IQR 20.03)	Correlation to Change Factor AHI (0.58; IQR 0.64)	Correlation to ESS (8; IQR 12)
SWV sGH oSTIM (2.11 m/s, IQR 0.57)	−0.40 (*p* = 0.874; corr. *p* ≥ 0.999)	0.102 (*p* = 0.687; corr. *p* ≥ 0.999)	−0.350 (*p* = 0.155; corr. *p* ≥ 0.999)
SWV sGH wSTIM (2.53 m/s, IQR 1.5)	−0.234 (*p* = 0.349; corr. *p* ≥ 0.999)	−0.414 (*p* = 0.088; corr. *p* ≥ 0.999)	0.121 (*p* = 0.631; corr. *p* ≥ 0.999)
DIFF SWV sGH wSTIM-oSTIM (0.45 m/s, IQR 1.89)	−0.174 (*p* = 0.489; corr. *p* ≥ 0.999)	−0.373 (*p* = 0.128; corr. *p* ≥ 0.999)	0.262 (*p* = 0.293; corr. *p* ≥ 0.999)
SWV sGG oSTIM (2.50 m/s, IQR 0.96)	0.104 (*p* = 0.681; corr. *p* ≥ 0.999)	0.232 (*p* = 0.354; corr. *p* ≥ 0.999)	−0.252 (*p* = 0.313; corr. *p* ≥ 0.999)
SWV sGG wSTIM (4.78 m/s, IQR 1.32)	−0.092 (*p* = 0.717; corr. *p* ≥ 0.999)	−0.323 (*p* = 0.191; corr. *p* ≥ 0.999)	0.111 (*p* = 0.661; corr. *p* ≥ 0.999)
DIFF SWV sGG wSTIM-oSTIM (2.16 m/s; IQR 1.39)	−0.074 (*p* = 0.769; corr. *p* ≥ 0.999)	−0.385 (*p* = 0.115; corr. *p* ≥ 0.999)	0.290 (*p* = 0.244; corr. *p* ≥ 0.999)
SWV nGH oSTIM (2.03 m/s, IQR 0.73)	−0.469 (*p* = 0.050; corr. *p* = 0.7)	−0.343 (*p* = 0.164; corr. *p* ≥ 0.999)	−0.115 (*p* = 0.650; corr. *p* ≥ 0.999)
SWV nGH wSTIM (1.69 m/s; IQR 0.48)	**−0.622 (*p* = 0.006**; corr. *p* = 0.096)	−0.420 (*p* = 0.083; corr. *p* ≥ 0.913)	−0.291 (*p* = 0.241; corr. *p* ≥ 0.999)
DIFF SWV nGH wSTIM-oSTIM (−0.16 m/s; IQR 0.53)	−0.053 (*p* = 0.836; corr. *p* ≥ 0.999)	−0.083 (*p* = 0.880; corr. *p* ≥ 0.999)	−0.052 (*p* = 0.838; corr. *p* ≥ 0.999)
SWV nGG oSTIM (2.48 m/s, IQR 0.62)	−0.045 (*p* = 0.858; corr. *p* ≥ 0.999)	0.035 (*p* = 0.890; corr. *p* ≥ 0.999)	0.415 (*p* = 0.087; corr. *p* ≥ 0.999)
SWV nGG wSTIM (2.21 m/s; 1.06)	−0.426 (*p* = 0.078; corr. *p* ≥ 0.63)	−0.169 (*p* = 0.502; corr. *p* ≥ 0.999)	−0.052 (*p* = 0.838; corr. *p* ≥ 0.999)
DIFF SWV nGG wSTIM-oSTIM (−0.43 m/s; IQR 1.16)	−0.358 (*p* = 0.145; corr. *p* ≥ 0.999)	−0.170 (*p* = 0.499 corr. *p* ≥ 0.999)	−0.287 (*p* = 0.248; corr. *p* ≥ 0.999)
Change factor SWV sGH wSTIMoSTIM (1.24; IQR 0.81)	−0.191 (*p* = 0.448; corr. *p* ≥ 0.999)	−0.393 (*p* = 0.106 corr. *p* ≥ 0.999)	0.285 (*p* = 0.251; corr. *p* ≥ 0.999)
Change factor SWV sGG wSTIMoSTIM (1.82; IQR 0.64)	−0.057 (*p* = 0.823; corr. *p* ≥ 0.999)	−0.323 (*p* = 0.191 corr. *p* ≥ 0.999)	0.315 (*p* = 0.202; corr. *p* ≥ 0.999)
Change factor SWV nGH wSTIMoSTIM (0.93; IQR 0.25)	−0.077 (*p* = 0.760; corr. *p* ≥ 0.999)	0.034 (*p* = 0.893 corr. *p* ≥ 0.999)	−0.074 (*p* = 0.77; corr. *p* ≥ 0.999))
Change factor SWV nGG wSTIMoSTIM (0.82; IQR 0.51)	**−0.484 (*p* = 0.042**; corr. *p* ≥ 0.63)	−0.294 (*p* = 0.236 corr. *p* ≥ 0.999)	−0.315 (*p* = 0.202; corr. *p* ≥ 0.999)

Abbreviations: shear wave velocity (SWV), musculus geniohyoideus at the stimulation side (sGH), musculus geniohyoideus at the non-stimulated side (nGH), musculus genioglossus at the stimulation side (sGG), musculus genioglossus at the non-stimulated side (nGG), with stimulation (wSTIM), without stimulation (oSTIM), difference (DIFF), Apnoe-Hypopnoe Index (AHI), Epworth Sleepiness Scale (ESS), corrected *p*-value using Bonferroni–Holm’s method (corr. *p*).

**Table 4:** Overview and comparison of clinical results due to tongue protrusion (Mann–Whitney U-test).

	Bilateral Tongue Protrusion (*n* = 9)	Contralateral Tongue Protrusion (*n* = 8)	
ESS	5 (IQR 5)	14.5 (IQR 12)	*p* = 0.1
Baseline AHI	26.4/h (IQR 36.3)	14.5 (IQR 12)	*p* = 0.773
Current AHI under therapy	14.5/h (IQR 13.05)	15.1/h (IQR 31.43)	*p* = 0.847
Change factor AHI	0.7932 (IQR 0.54)	0.5587 (IQR 0.81)	*p* = 0.7
Therapy success AHI < 15/h	*n* = 5	*n* = 4	*p* = 0.824

Abbreviations: Apnoe–Hypopnoe Index (AHI), Epworth Sleepiness Scale (ESS).

**Table 5:** Evaluation of SWV data separated by tongue protrusion pattern with regard to therapy success (Mann–Whitney U-test).

	Therapy Success AHI < 15/h (Yes vs. No) (Mann–Whitney U Test)	
	Bilateral Tongue Protrusion (*n* = 9)	Contralateral Tongue Protrusion (*n* = 8)
SWV sGH oSTIM (m/s)	2.25 (IQR 0.63) vs. 2.26 (IQR 0.91); *p* = 1.000 (corr. *p* ≥ 0.999)	2.05 (IQR 0.59) vs. 2.18 (IQR 0.52); *p* = 0.486 (corr. *p* ≥ 0.999)
SWV sGH wSTIM (m/s)	2.26 (IQR 1.14) vs. 2.76 (IQR 1.95); *p* = 0.730 (corr. *p* ≥ 0.999)	3.22 (IQR 1.08) vs. 2.42 (IQR 1.93); *p* = 0.343 (corr. *p* ≥ 0.999)
SWV sGG oSTIM (m/s)	2.99 (IQR 0.88) vs. 2.40 (IQR 0.96); *p* = 0.286 (corr. *p* ≥ 0.999)	**2.08 (IQR 0.64) vs. 2.97 (IQR 0.51); *p* = 0.029** (corr. *p* = 0.348)
SWV sGG wSTIM (m/s)	5.16 (IQR 1.95) vs. 4.52 (IQR 1.31); *p* = 1.000 (corr. *p* ≥ 0.999)	4.53 (1.89) vs. 4.78 (IQR 1.47); *p* = 1.000 (corr. *p* ≥ 0.999)
SWV nGH oSTIM (m/s)	2.15 (IQR 0.4) vs. 1.78 (IQR 1.03); *p* = 0.286 (corr. *p* ≥ 0.999)	2.29 (IQR 0.58) vs. 1.68 (IQR 1.05); *p* = 0.343 (corr. *p* ≥ 0.999)
SWV nGH wSTIM (m/s)	2.01 (IQR 0.66) vs. 1.96 (IQR 0.98); *p* = 0.413 (corr. *p* ≥ 0.999)	2.12 (IQR 0.56) vs. 1.65 (IQR 0.32); *p* = 0.114 (corr. *p* ≥ 0.999)
SWV nGG oSTIM (m/s)	2.44 (IQR 0.79) vs. 2.26 (IQR 1.81); *p* = 0.905 (corr. *p* ≥ 0.999)	2.72 (IQR 0.36) vs. 2.56 (IQR 0.71); *p* = 0.686 (corr. *p* ≥ 0.999)
SWV nGG wSTIM (m/s)	2.16 (IQR 2.45) vs. 2.31 (IQR 1.31); *p* = 0.730 (corr. *p* ≥ 0.999)	2.39 (IQR 1.61) vs. 1.66 (IQR 0.99); *p* = 0.200 (corr. *p* ≥ 0.999)
Change Factor SWV sGH wSTIMoSTIM	1.03 (IQR 0.56) vs. 1.17 (IQR 1.11); *p* = 1.000 (corr. *p* ≥ 0.999)	1.62 (IQR 1.05) vs. 1.04 (IQR 1.12; *p* = 0.343 (corr. *p* ≥ 0.999)
Change Factor SWV sGG wSTIMoSTIM	**1.70 (IQR 0.27) vs. 1.98 (IQR 0.75); *p* = 0.032** (corr. *p* = 0.384)	2.31 (IQR 1.50) vs. 1.63 (IQR 0.76); *p* = 0.343 (corr. *p* ≥ 0.999)
Change Factor SWV nGH wSTIMoSTIM	0.93 (IQR 0.46) vs. 0.98 (IQR 0.27); *p* = 0.730 (corr. *p* ≥ 0.999)	0.95 (IQR 0.41) vs. 0.91 (IQR 0.44); *p* = 0.886 (corr. *p* ≥ 0.999)
Change Factor SWV nGG wSTIMoSTIM	1.23 (IQR 0.95) vs. 0.96 (IQR 0.63); *p* = 0.286 (corr. *p* ≥ 0.999)	0.94 (IQR 0.57) vs. 0.68 (IQR 0.48); *p* = 0.343 (corr. *p* ≥ 0.999)

Abbreviations: shear wave velocity (SWV), musculus geniohyoideus at the stimulation side (sGH), musculus geniohyoideus at the non-stimulated side (nGH), musculus genioglossus at the stimulation side (sGG), musculus genioglossus at the non-stimulated side (nGG), with stimulation (wSTIM), without stimulation (oSTIM), Apnoe–Hypopnoe Index (AHI), corrected *p*-value using Bonferroni–Holm’s method (corr. *p*).

**Table 6:** Spearman correlations between SWV data of patients with bilateral tongue protrusion and clinical therapy outcome parameters.

Bilateral Tongue Protrusion (*n* = 9)			
	Correlation with Current AHI under Therapy	Correlation with Change Factor AHI	Correlation with ESS
SWV sGH oSTIM (m/s)	−0.150 (*p* = 0.700; corr. *p* ≥ 0.999)	−0.050 (*p* = 0.898; corr. *p* ≥ 0.999)	−0.017 (*p* = 0.965; corr. *p* ≥ 0.999)
SWV sGH wSTIM (m/s)	0.017 (*p* = 0.966; corr. *p* ≥ 0.999)	−0.367 (*p* = 0.332; corr. *p* ≥ 0.999)	0.051 (*p* = 0.897; corr. *p* ≥ 0.999)
SWV sGG oSTIM (m/s)	−0.633 (*p* = 0.067; corr. *p* ≥ 0.804)	−0.517 (*p* = 0.154; corr. *p* ≥ 0.999)	−0.475 (*p* = 0.197; corr. *p* ≥ 0.999)
SWV sGG wSTIM (m/s)	−0.217 (*p* = 0.576; corr. *p* ≥ 0.999)	−0.383 (*p* = 0.308; corr. *p* ≥ 0.999)	−0.085 (*p* = 0.828; corr. *p* ≥ 0.999)
SWV nGH oSTIM (m/s)	−0.502 (*p* = 0.168; corr. *p* ≥ 0.999)	−0.393 (*p* = 0.295; corr. *p* ≥ 0.999)	0.043 (*p* = 0.913; corr. *p* ≥ 0.999)
SWV nGH wSTIM (m/s)	−0.517 (*p* = 0.154; corr. *p* ≥ 0.999)	−0.350 (*p* = 0.356; corr. *p* ≥ 0.999)	−0.271 (*p* = 0.480; corr. *p* ≥ 0.999)
SWV nGG oSTIM (m/s)	−0.192 (*p* = 0.620; corr. *p* ≥ 0.999)	0.042 (*p* = 0.915; corr. *p* ≥ 0.999)	0.366 (*p* = 0.333; corr. *p* ≥ 0.999)
SWV nGG wSTIM (m/s)	−0.418 (*p* = 0.262; corr. *p* ≥ 0.999)	0.075 (*p* = 0.847; corr. *p* ≥ 0.999)	−0.289 (*p* = 0.450; corr. *p* ≥ 0.999)
Change Factor SWV sGH wSTIMoSTIM	0.177 (*p* = 0.765; corr. *p* ≥ 0.999)	−0.250 (*p* = 0.516; corr. *p* ≥ 0.999)	0.186 (*p* = 0.631; corr. *p* ≥ 0.999)
Change Factor SWV sGG wSTIMoSTIM	0.550 (*p* = 0.125; corr. *p* ≥ 0.999)	0.250 (*p* = 0.516; corr. *p* ≥ 0.999)	0.220 (*p* = 0.569; corr. *p* ≥ 0.999)
Change Factor SWV nGH wSTIMoSTIM	0.033 (*p* = 0.932; corr. *p* ≥ 0.999)	0.267 (*p* = 0.488; corr. *p* ≥ 0.999)	−0.237 (*p* = 0.539; corr. *p* ≥ 0.999)
Change Factor SWV nGG wSTIMoSTIM	−0.533 (*p* = 0.139; corr. *p* ≥ 0.999)	−0.183 (*p* = 0.637; corr. *p* ≥ 0.999)	−0.593 (*p* = 0.092; corr. *p* ≥ 0.999)

Abbreviations: shear wave velocity (SWV), musculus geniohyoideus at the stimulation side (sGH), musculus geniohyoideus at the non-stimulated side (nGH), musculus genioglossus at the stimulation side (sGG), musculus genioglossus at the non-stimulated side (nGG), with stimulation (wSTIM), without stimulation (oSTIM), Apnoe–Hypopnoe Index (AHI), Epworth Sleepiness Scale (ESS), corrected *p*-value using Bonferroni–Holm’s method (corr. *p*).

**Table 7:** Spearman correlations between SWV data of patients with contralateral tongue protrusion and clinical therapy outcome parameters.

Contralateral Tongue Protrusion (*n* = 8)			
	**Correlation with Current AHI under Therapy**	Correlation with Change Factor AHI	Correlation with ESS
SWV sGH oSTIM (m/s)	0.071 (*p* = 0.867; corr. *p* ≥ 0.999)	0.357 (*p* = 0.385; corr. *p* ≥ 0.999)	−0.539 (*p* = 0.168; corr. *p* ≥ 0.999)
SWV sGH wSTIM (m/s)	−0.262 (*p* = 0.531; corr. *p* ≥ 0.999)	−0.452 (*p* = 0.260; corr. *p* ≥ 0.999)	0.335 (*p* = 0.417; corr. *p* ≥ 0.999)
SWV sGG oSTIM (m/s)	**0.714 (*p* = 0.047**; corr. *p* = 0.564)	**0.810 (*p* = 0.015**; corr. *p* = 0.18)	−0.192 (*p* = 0.649; corr. *p* ≥ 0.999)
SWV sGG wSTIM (m/s)	0.214 (*p* = 0.610; corr. *p* ≥ 0.999)	0.048 (*p* = 0.911; corr. *p* ≥ 0.999)	0.371 (*p* = 0.365; corr. *p* ≥ 0.999)
SWV nGH oSTIM (m/s)	−0.500 (*p* = 0.207; corr. *p* ≥ 0.999)	−0.214 (*p* = 0.610; corr. *p* ≥ 0.999)	−0.240 (*p* = 0.568; corr. *p* ≥ 0.999)
SWV nGH wSTIM (m/s)	−0.690 (*p* = 0.058; corr. *p* = 0.638)	−0.619 (*p* = 0.102; corr. *p* ≥ 0.999)	−0.287 (*p* = 0.490; corr. *p* ≥ 0.999)
SWV nGG oSTIM (m/s)	−0.190 (*p* = 0.651; corr. *p* ≥ 0.999)	0.167 (*p* = 0.693; corr. *p* ≥ 0.999)	0.048 (*p* = 0.910; corr. *p* ≥ 0.999)
SWV nGG wSTIM (m/s)	−0.381 (*p* = 0.352; corr. *p* ≥ 0.999)	−0.286 (*p* = 0.493; corr. *p* ≥ 0.999)	0.156 (*p* = 0.713; corr. *p* ≥ 0.999)
Change Factor SWV sGH wSTIMoSTIM	−0.286 (*p* = 0.493; corr. *p* ≥ 0.999)	−0.476 (*p* = 0.233; corr. *p* ≥ 0.999)	0.299 (*p* = 0.471; corr. *p* ≥ 0.999)
Change Factor SWV sGG wSTIMoSTIM	−0.310 (*p* = 0.456; corr. *p* ≥ 0.999)	−0.524 (*p* = 0.183; corr. *p* ≥ 0.999)	0.287 (*p* = 0.490; corr. *p* ≥ 0.999)
Change Factor SWV nGH wSTIMoSTIM	0.048 (*p* = 0.911; corr. *p* ≥ 0.999)	−0.214 (*p* = 0.610; corr. *p* ≥ 0.999)	0.275 (*p* = 0.509; corr. *p* ≥ 0.999)
Change Factor SWV nGG wSTIMoSTIM	−0.310 (*p* = 0.456; corr. *p* ≥ 0.999)	−0.333 (*p* = 0.420; corr. *p* ≥ 0.999)	0.132 (*p* = 0.756; corr. *p* ≥ 0.999)

Abbreviations: shear wave velocity (SWV), musculus geniohyoideus at the stimulation side (sGH), musculus geniohyoideus at the non-stimulated side (nGH), musculus genioglossus at the stimulation side (sGG), musculus genioglossus at the non-stimulated side (nGG), with stimulation (wSTIM), without stimulation (oSTIM), Apnoe–Hypopnoe Index (AHI), Epworth Sleepiness Scale (ESS), corrected *p*-value using Bonferroni–Holm’s method (corr. *p*).

## Data Availability

The data presented in this study are available on request from the corresponding author. The evaluation refers to available ultrasound data of the presented patient cohort. A detailed presentation of the ultrasound data is published under “Arens, P.; Fischer, T.; Dommerich, S.; Olze, H.; Lerchbaumer, M.H. Ultrasound Shear Wave Elastography of the Tongue during Selective Hypoglossal Nerve Stimulation in Patients with Obstructive Sleep Apnea Syndrome. *Ultrasound Med. Biol.* **2021**, *47*, 2869–2879, https://doi.org/10.1016/J.ULTRASMEDBIO.2021.06.009” [16].
